# Study of the Effects of Recognition of Stress on Symptoms and Regular Hospital Visits: An Analysis from Japanese National Statistics

**DOI:** 10.3390/healthcare8030274

**Published:** 2020-08-14

**Authors:** Akira Komatsuzaki, Sachie Ono

**Affiliations:** Department of Preventive and Community Dentistry, The Nippon Dental University, School of Life Dentistry at Niigata, Niigata 951-8151, Japan; sachie@ngt.ndu.ac.jp

**Keywords:** stress, health behavior, symptom assessment, disease, quality of life

## Abstract

Stress is a risk factor for numerous lifestyle diseases, including dental diseases. The purpose of the present study was to investigate how sensitivity to psychological stress relates to subjective symptoms and regular hospital visits using information from the large-scale database of national statistics. Anonymized data from 10,584 respondents aged 30–79 of the Japanese 2013 Comprehensive Survey of Living Conditions were analyzed. Respondents were classified by age into a middle-aged group and an elderly group, and a contingency table analysis, rank correlation analysis, and binomial logistic regression analysis were performed. Contingency table analysis confirmed that eight items were related to the presence of a recognition of stress, including the presence of subjective symptoms (*p* < 0.001), the presence of regular hospital visits (*p* < 0.001), symptoms of periodontal disease (*p* < 0.001), and self-rated health (*p* < 0.001). Responses for symptoms and diseases requiring regular hospital visits were ranked in order for a stress group and a no stress group, and it was found that other than fatigue symptoms being ranked highly in the stress group, there were no particular differences. Logistic regression analysis results showed significant odds ratios for six items including: self-rated health (3.91, 95% CI, 3.23 to 4.73), lifestyle awareness (1.96, 95% CI, 1.68 to 2.28), and symptoms of periodontal disease (1.71, 95% CI 1.19 to 2.48). The present study showed that susceptibility to psychological stress is related to awareness of subjective symptoms and to regular hospital visits due to disease, suggesting that these have direct and indirect mutual effects.

## 1. Introduction

It has previously been pointed out that the recognition and evaluation of symptoms vary greatly according to individual subjectivity, and that for this reason some diseases are more susceptible to the effects of psychological stress than others [1].

In Japan, the increase in work-related injury or death as a result of overwork led to the start of the Stress Check Program in 2015, which uses questionnaires to check stress [2]. The stress check questionnaire includes a number of questions related to subjective symptoms and regular hospital visits and was created based on prior evidence showing that stress is related to somatic symptoms and to the onset and progression of disease [3].

At the same time, it has been shown that somatic symptoms and regular hospital visits for treatment of disease are affected by psychological state, and a report by Larson et al. [4] on subjective symptom management capability pointed to the importance of improving the symptom management capability of individuals in order to improve their quality of life (QOL), and proposed evaluation models including the Model of Symptom Management (MSM). These are mainly used in the field of nursing and focus on the effects of psychological factors on consultation behavior.

In addition, there have been numerous reports of an association between stress and lifestyle [5,6,7], and in countries where there is a need to focus on measures for lifestyle-related diseases, it is important to clarify the stress factors that are part of the backdrop to disease.

This study evaluated how sensitivity to psychological stress relates to subjective symptoms and regular hospital visits using the large-scale national statistics database.

## 2. Materials and Methods

### 2.1. Study Population

Anonymized data sheets of the questionnaires (household survey, health survey, in Japanese) from the 2013 Comprehensive Survey of Living Conditions were obtained following the Ministry of Health, Labour and Welfare’s access to data process. The data from 10,584 individuals in the 30–79 age group (5092 men, 5492 women) were used for the analysis. The subjects were limited to people aged 30 or over because the Survey of Dental Diseases conducted at the same time found this to be the period during which the proportion of people with missing teeth increases, and also the government considers this to be the target age group for health checkups and health guidance. The subjects were grouped into two age groups: the middle-aged group (30–59 years) and the elderly group (60–79 years).

### 2.2. Study Design

**Figure 1:**
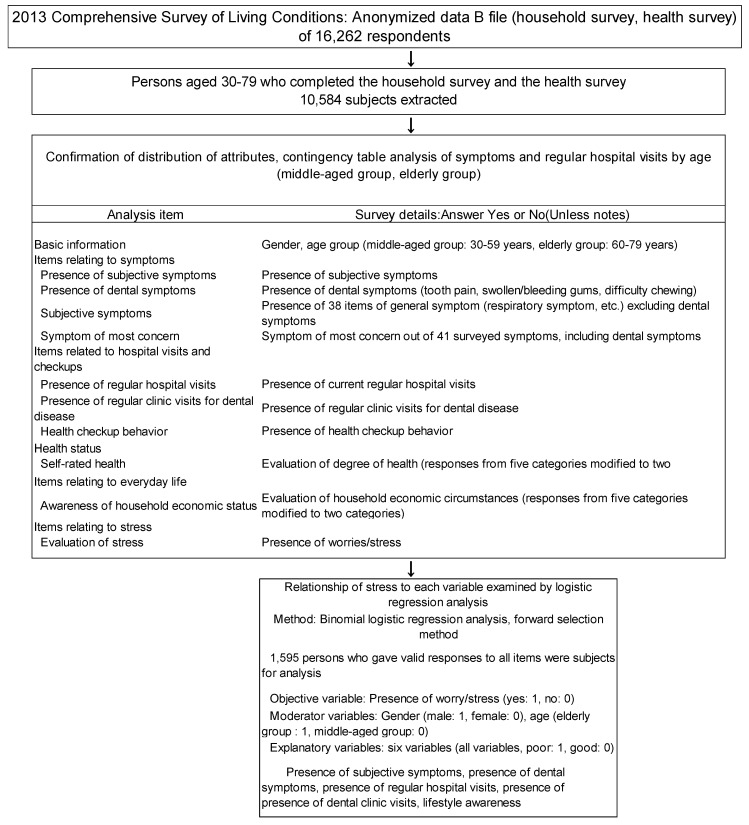
Outline of the data analysis in the study.

In the present study, a stepwise analysis was performed according to the design shown in Figure 1 in order to explore and clarify the characteristics of factors relating to stress from the results of a large-scale cross-sectional survey.

In the first step, a contingency table analysis of age group (middle-aged/elderly) and recognition of worry/stress (yes/no) was performed. Trends in the frequency of response for symptoms and diseases were compared by rank correlation and mean rank difference.

The items used for analysis were items from the Comprehensive Survey of Living Conditions, including subjective symptoms, disease requiring regular hospital visits, lifestyle awareness, self-rated health, recognition of worry/stress, and behavior with respect to public health checkups.

For the second step, a binomial logistic regression analysis of subjects who answered all questions (*n* = 1595) was performed, with stress as the objective variable and items shown from the contingency table analysis to be related to stress as explanatory variables.

### 2.3. Statistical Analysis

For aggregate analysis, Microsoft Excel 2010 (Microsoft Japan Co., Ltd., Tokyo, Japan) and Excel-Toukei 2012 (Social Survey Research Information Co. Ltd., Tokyo, Japan) were used. The tests used for statistically significant differences were the χ^2^ test for the contingency table analysis, Spearman’s rank correlation coefficient for the rank correlation analysis, and the Friedman test for mean rank difference. The binomial logistic regression analysis used the partial correlation coefficient test for significance, and the level of significance was set at *p* < 0.05 for all tests.

### 2.4. Ethical Considerations

The data analyzed in the present study were the results of a national survey carried out in line with the Japanese regulations on surveys and were processed for anonymization by the Ministry of Health, Labour and Welfare. Permission to conduct the study was obtained in accordance with the provisions of Article 36 of the Japanese Statistics Act. All subjects gave their informed consent for inclusion before they participated in the study. The study was conducted in accordance with the Declaration of Helsinki, and the protocol was approved by the Ethics Committee of School of Life Dentistry at Niigata, the Nippon Dental University (approval no. ECNG-R-398).

## 3. Results

The middle-aged group, which covered a wide age range, accounted for the majority of subjects (57.3%). In both the middle-aged group and the elderly group, there were slightly more women than men (Table 1).

Table 2 shows the responses to questionnaire items by age group. Subjects who were aware of stress accounted for the majority (54.2%) of the middle-aged group but for only 40.5% of the elderly group, and this difference was significant (*p* < 0.001).

The proportion of subjects with subjective symptoms and making regular hospital visits was significantly greater in the elderly group than in the middle-aged group (*p* < 0.001).

In the items related to dentistry, the number of subjects with symptoms of difficulty chewing was significantly greater in the elderly group, and the number of subjects with regular dental clinic visits was significantly greater in the middle-aged group (both *p* < 0.001).

Overall, with the exception of drinking alcohol and the two dental symptoms, there were significant differences between the two age groups for all items.

Table 3 shows the symptoms with highest frequency of response (ranked in order up to the 10th) by stress group.

Chronic musculoskeletal system symptoms, which are common from middle age onward, ranked highest in both groups, and “feeling listless”, which may be conjectured to be greatly affected by fatigue, ranked high in the stress group. Spearman’s rank correlation coefficient obtained by ranking the frequency of responses for all symptoms in the two groups was high at 0.851, but a significant difference was found in the comparison of mean rank (*p* < 0.01).

Table 4 shows the diseases requiring regular hospital visits, with highest frequency of response (ranked in order up to the 10th) by stress group.

High blood pressure and dyslipidemia, which are typical lifestyle diseases, were highest in both groups. Regular clinic visits for dental disease were ranked 5th in both groups, and the ranking tended to be similar in both groups.

In the stress group, depression and other mental diseases were ranked in the top 10 diseases. Spearman’s rank correlation coefficient from the frequency of response for all diseases requiring regular hospital visits in the two groups was high at 0.923, but a significant difference was found in the comparison of mean rank (*p* < 0.001).

Table 5 shows the responses to the survey items by stress group. There were significant differences between the groups for all items with the exception of two dental symptoms and smoking. Among the items for which significant differences were found, items with a particularly high proportion in the stress group were perception of health (poor), which was approximately 82%, and swollen/bleeding gums (yes), which was 78.7%. Presence of subjective symptoms (yes) also accounted for approximately 70% of the stress group (*p* < 0.001).

Table 6 shows the results of the logistic regression analysis. Six explanatory variables were selected, and the highest odds ratios were, in order, perception of health (3.91, 95% confidence interval (CI): 3.23–4.73), lifestyle awareness (1.96, 95% CI: 1.68–2.28), and periodontal disease symptoms (1.71, 95% CI: 1.19–2.48). The coefficient of determination *R*^2^, indicating the accuracy of the analysis, was 0.176, and the percentage of correct classifications was 69.9%.

## 4. Discussion

Workplace stress is widely recognized at the global level to be an issue of concern that affects not just the health of employees but also the productivity of companies [8].

The World Health Organization (WHO) has published a report showing the effects caused by psychological burden at the workplace, which notes a tendency for people with work-related stress to be susceptible to physical fatigue and insomnia, as well as to diseases including heart disease, indigestion, high blood pressure, headaches, and musculoskeletal system disorders such as lumbago [9].

The results of the present study also showed that conditions such as feeling listless were common in the stress group, suggesting that there are certain symptoms or diseases that are specifically susceptible to the effects of stress.

As informatization advances around the world, there has been increasing research into the effects of stressful life events [10]. This led to the WHO publishing its comprehensive Mental Health Action Plan 2013–2020 in 2013 [11], which aimed to promote mental well-being under the principle of “no health without mental health”.

In Japan, depression/mental illness is one of the many diseases for which community-based healthcare measures are being reinforced and promoted [12]. In 2017, the number of patients in Japan with psychosis (mental diseases, behavioral impairment) reached 2.7 per 100 population [13]. Measures for stress aimed at preventing depression and other mental diseases have thus become a major mental health challenge in Japan.

Given this situation, legislation was passed in 2015 making stress checks for employees mandatory, and stress evaluations by means of questionnaire forms have become a familiar occurrence [2].

There have already been numerous reports on the association between stress and somatic symptoms or disease [14,15,16], which have included dental diseases such as periodontal disease and temporomandibular joint disorder [17,18].

The idea that stress should be considered a cause or a risk factor for disease has already spread at the global level, and this is reflected in the International Classification of Diseases (ICD) of the WHO [19] and the Diagnostic and Statistical Manual of Mental Disorders of the American Psychiatric Association [20]. In the most recent ICD (ICD-11), the disease classification of “disorders specifically associated with stress” is listed. It is important to consider a survey method that covers the general population and is universal.

In a prior study investigating the relationship between pain and stress, Abdallah et al. [21] found a relationship between chronic stress and chronic pain, pointing out that the continuation of stress or pain causes physical maladjustments that may lead to reduced levels of health. In the results of the present study, headache was included among the higher ranked symptoms in the stress group.

The present study included a group of elderly subjects, and palliative care is becoming increasingly important in Japan as society becomes ever more long-lived. Evaluation models such as the MSM [4], which focuses on the significance of the coping behaviors of individuals with respect to symptoms, are increasingly being used and are contributing to improved QOL.

Prevention of lifestyle diseases from entry into adulthood onward is an important and necessary measure for extending healthy life expectancy [13]. In Japan, overlapping risk factors such as smoking, alcohol, and underlying diseases [22] is viewed as a problem, and measures to address health disparities are being promoted nationwide [23].

In addition, the present results have also indicated a relationship between dental symptoms and stress, and there is a need in future health guidance to build linkages between the fields of oral health and mental health.

In the present study, the proportion of subjects making regular clinic visits was higher than the proportion of subjects with the three dental diseases. There is a need to understand the process of symptom management that leads to dental consultation behavior, and it is therefore important to analyze the background information, such as the period of appearance of symptoms and the precise details. Stress has been reported to be a factor impeding the treatment of diseases [24], and the results of the present logistic regression analysis with stress as the objective variable showed an odds ratio of 1.48 (95% CI: 1.23 to 1.79) for not having health checkups, suggesting the possibility that stress may affect healthcare.

A limitation of the present study is that it used data from the Comprehensive Survey of Living Conditions, which only gives a cross-sectional view of short-term symptoms reported from the “past few days.” This makes it difficult to gain a picture of symptoms that can readily disappear over a short period. In addition, it has been pointed out that there are many limits to evaluating stress by means of a questionnaire [25]. Because of limits to the number of options, insomnia, which has been identified as having a relationship to stress [26], was not included among the options for symptoms. A more objective method for the evaluation of stress is to obtain samples of saliva and blood, and to examine these for stress markers [27,28,29]. However, from the standpoint of simplicity and economy, this method cannot easily be used in a large-scale survey.

## 5. Conclusions

Using anonymized data of adults from the 2013 Comprehensive Survey of Living Conditions, the relationship between symptoms and regular hospital visits was investigated in stress and no stress groups.

The present study confirmed the relationship between recognition of stress, subjective symptoms, and hospital visits. However, it will be necessary in future work to improve the method of stress evaluation in order to investigate the effects of stress on self-rated health and on everyday life.

These results suggest the possibility that recognition of psychological stress may have direct and indirect effects on the awareness of subjective symptoms and contracting disease.

## Figures and Tables

**Table 1:** Number of persons whose anonymized data were used.

Gender	Age Group					
	Middle-Aged (30–59 Years)		Elderly (60–79 Years)		Total	
Male	2925	(48.2)	2167	(48.0)	5092	(48.1)
Female	3143	(51.8)	2349	(52.0)	5492	(51.9)
Total	6068	(100.0)	4516	(100.0)	10,584	(100.0)

Values are presented as numbers with percentages in parentheses.

**Table 2:** Responses by subject attributes.

Attributes	Middle-Aged Group	(%)	Elderly Group	(%)	Total	(%)	*p* Value (χ^2^ Test)
Presence of worries/stress							
Yes	3286	54.2	1829	40.5	5115	48.3	<0.001
No	2782	45.8	2687	59.5	5469	51.7	
Presence of subjective symptoms							
Yes	1758	29.0	1895	42.0	3653	34.5	<0.001
No	4310	71.0	2621	58.0	6931	65.5	
Symptoms: tooth pain ^a^							
Yes	114	6.5	97	5.1	211	5.8	0.077
No	1644	93.5	1798	94.9	3442	94.2	
Symptoms: swollen/bleeding gums ^a^							
Yes	104	5.9	140	7.4	244	6.7	0.075
No	1654	94.1	1755	92.6	3409	93.3	
Symptoms: difficulty chewing ^a^							
Yes	51	2.9	154	8.1	205	5.6	<0.001
No	1707	97.1	1741	91.9	3448	94.4	
Presence of regular hospital visits							
Yes	1844	30.4	2991	66.2	4835	45.7	<0.001
No	4219	69.5	1523	33.7	5742	54.3	
Presence of regular dental clinic visits ^b^							
Yes	278	15.1	324	10.8	602	12.5	<0.001
No	1566	84.9	2667	89.2	4233	87.5	
Self-rated health							
Poor	648	10.7	820	18.2	1468	13.9	<0.001
Regular/good	5420	89.3	3696	81.8	9116	86.1	
Smoking							
Yes	1672	27.6	698	15.5	2370	22.4	<0.001
No	4396	72.4	3818	84.5	8214	77.6	
Drinking alcohol							
Yes	2005	33.0	1450	32.1	3455	32.6	0.311
No	4063	67.0	3066	67.9	7129	67.4	
Health checkup behavior							
No checkups	1851	30.5	1633	36.2	3484	32.9	<0.001
Checkups	4217	69.5	2883	63.8	7100	67.1	
Lifestyle awareness							
Harsh	3830	63.1	2683	59.4	6513	61.5	<0.001
Regular/comfortable	2238	36.9	1833	40.6	4071	38.5	
Total	6068	(100.0)	4516	(100.0)	10,584	(100.0)	

**^a^** Proportion of subjects to those with subjective symptoms. **^b^** Proportion of subjects to those making regular hospital visits.

**Table 3:** Symptoms with highest frequency of response by stress group (top 10 ranked).

**Stress Group**	**1st**	**2nd**	**3rd**	**4th**	**5th**	**6th**	**7th**	**8th**	**9th**	**10th**
No worries/stress group	Lower back pain	Stiff shoulders	Joint pain in hands and feet	Cough/phlegm	Blocked nose/nasal discharge	Numbness of limbs	Itchy eyes	Tinnitus	Frequent urination	Difficulty hearing
Number (%)	405 (7.4)	309 (5.7)	213 (3.9)	141 (2.6)	131 (2.4)	122 (2.2)	121 (2.2)	108 (2.0)	107 (2.0)	106 (1.9)
	**1st**	**2nd**	**3rd**	**4th**	**5th**	**6th**	**7th**	**8th**	**9th**	**10th**
Worries/stress group	Lower back pain	Stiff shoulders	Feeling listless	Joint pain in hands and feet	Blurred vision	Headache	Cough/phlegm	Numbness of limbs	Itching	Blocked nose/ nasal discharge
Number (%)	983 (19.2)	934 (18.3)	499 (9.8)	479 (9.4)	406 (7.9)	368 (7.2)	363 (7.1)	354 (6.9)	338 (6.6)	336 (6.6)

**Table 4:** Diseases for which there were regular hospital visits with highest frequency of response by stress group (top 10 ranked).

**Stress Group**	**1st**	**2nd**	**3rd**	**4th**	**5th**	**6th**	**7th**	**8th**	**9th**	**10th**
No worries/stress group	High blood pressure	Dyslipidemia	Diabetes	Eye disease	Dental disease	Lumbago	Stiff shoulders	Gastroduodenal disease	Angina/cardiac infarction	Prostatic hyperplasia
Number (%)	834 (15.3)	335 (6.1)	325 (5.9)	281 (5.1)	262 (4.8)	250 (4.6)	125 (2.3)	107 (2.0)	93 (1.7)	87 (1.6)
	**1st**	**2nd**	**3rd**	**4th**	**5th**	**6th**	**7th**	**8th**	**9th**	**10th**
Worries/stress group	High blood pressure	Dyslipidemia	Lumbago	Diabetes	Dental disease	Eye disease	Stiff shoulders	Joint disease	Depression, etc.	Other skin disease
Number (%)	776 (15.2)	383 (7.5)	382 (7.5)	354 (6.9)	340 (6.7)	335 (6.6)	243 (4.8)	180 (3.5)	167 (3.3)	144 (2.8)

**Table 5:** Comparison of responses to survey items by presence of worries/stress, and unadjusted odds ratios.

Survey Attributes	Yes	(%)	No	(%)	Total	(%)	Unadjusted ORs	*p* Value (χ^2^ Test)
Gender								
Male	2257	(44.3)	2835	(55.7)	5092	(100.0)	0.733	<0.001
Female	2858	(52.0)	2634	(48.0)	5492	(100.0)		
Self-rated health								
Poor	1199	(81.7)	269	(18.3)	1468	(100.0)	5.918	<0.001
Regular/good	3916	(43.0)	5200	(57.0)	9116	(100.0)		
Presence of subjective symptoms								
Yes	2516	(68.9)	1137	(31.1)	3653	(100.0)	3.688	<0.001
No	2599	(37.5)	4332	(62.5)	6931	(100.0)		
Presence of regular hospital visits								
Yes	2611	(54.0)	2224	(46.0)	4835	(100.0)	1.522	<0.001
No	2500	(43.5)	3242	(56.5)	5742	(100.0)		
Presence of regular clinic visits for dental disease								
Yes	340	(56.5)	262	(43.5)	602	(100.0)	1.121	0.192
No	2271	(53.6)	1962	(46.4)	4233	(100.0)		
Symptoms: swollen/bleeding gums								
Yes	192	(78.7)	52	(21.3)	244	(100.0)	1.724	<0.001
No	2324	(68.2)	1085	(31.8)	3409	(100.0)		
Symptoms: difficulty chewing								
Yes	151	(73.7)	54	(26.3)	205	(100.0)	1.281	0.127
No	2365	(68.6)	1083	(31.4)	3448	(100.0)		
Symptoms: tooth pain								
Yes	2366	(68.7)	1076	(31.3)	3442	(100.0)	0.894	0.474
No	150	(71.1)	61	(28.9)	211	(100.0)		
Lifestyle awareness								
Harsh	3459	(53.1)	3054	(46.9)	6513	(100.0)	1.652	<0.001
Regular/comfortable	1656	(40.7)	2415	(59.3)	4071	(100.0)		
Smoking								
Yes	1134	(47.8)	1236	(52.2)	2370	(100.0)	0.976	0.595
No	3981	(48.5)	4233	(51.5)	8214	(100.0)		
Drinking alcohol								
Yes	1580	(45.7)	1875	(54.3)	3455	(100.0)	0.857	<0.001
No	3535	(49.6)	3594	(50.4)	7129	(100.0)		
Health checkup behavior								
No checkups	3374	(47.5)	3726	(52.5)	7100	(100.0)	0.907	0.017
Checkups	1741	(50.0)	1743	(50.0)	3484	(100.0)		

**Table 6:** Results of logistic regression analysis with presence of worries/stress as the objective variable.

Selected Explanatory Variable	Partial Regression Coefficient	Wald	*p* Value	Judgement	Odds Ratio	Lower Limit	Upper Limit
Self-rated health (1, poor/0, wrt: regular, good)	1.36	197.3	<0.001	**	3.91	3.23	4.73
Lifestyle awareness (1, harsh/0, wrt: regular, comfortable)	0.67	76.1	<0.001	**	1.96	1.68	2.28
Symptoms: swollen/bleeding gums (1, yes/0, wrt: no)	0.54	8.4	0.004	**	1.71	1.19	2.48
Symptoms: difficulty chewing (1, yes/0, wrt: no)	0.43	4.8	0.028	*	1.54	1.05	2.29
Health checkup behavior (1, no checkups/0, wrt: checkups)	0.39	17.2	<0.001	**	1.48	1.23	1.79
Age (1, elderly group/0, wrt: middle-aged group)	−0.67	73.0	<0.001	**	0.51	0.44	0.60

** *p* < 0.01, * *p* < 0.05. Only explanatory variables selected by the forward selection method are shown. *n* = 1595, coefficient of determination *R*^2^ = 0.176, percentage of correct classifications = 69.9%. wrt: with respect to.

## References

[1] McEwen B.S. (2017). Neurobiological and systemic effects of chronic stress. Chronic Stress (Thousand Oaks).

[2] Imamura K., Asai Y., Watanabe K. (2018). Effect of the National Stress Check Program on mental health among workers in Japan: A 1-year retrospective cohort study. J. Occup. Health.

[3] Kawakami N., Tsutsumi A. (2016). The Stress Check Program: A new national policy for monitoring and screening psychosocial stress in the workplace in Japan. J. Occup. Health.

[4] Larson P.J., Carrieri-Kohlman V., Dodd M.J., Douglas M., Faucett J., Froelicher E., Gortner S., Halliburton P., Janson S., Lee K.A. (1994). A model for symptom management. Image J. Nurs. Scholarsh..

[5] McEwen B.S., Stellar E. (1993). Stress and the individual. Mechanisms leading to disease. Arch. Int. Med..

[6] Turcu-Stiolica A., Subtirelu M.-S., Ciurea P.L., Cristian D.S., Bogdan M., Barbulescu A.L., Glavan D.-G., Turcu-Stiolica R.-A., Firulescu S.C., Chisalau B.A. (2020). The Influence of Socio-Demographic Factors, Lifestyle and Psychiatric Indicators on Adherence to Treatment of Patients with Rheumatoid Arthritis: A Cross-Sectional Study. Medicina.

[7] Ilacqua A., Izzo G., Emerenziani G.P., Baldari C., Aversa A. (2018). Lifestyle and fertility: The influence of stress and quality of life on male fertility. Reprod. Biol. Endocrinol..

[8] International Labour Office (2012). Stress Prevention at Work Checkpoints.

[9] World Health Organization (2003). Raising Awareness of Psychological Harassment at Work.

[10] Zorigt G., Enkh-Amgalan N., Yu T. (2019). Use of best-worst scaling to estimate the magnitude of stressful life events in older adults. Psychogeriatrics.

[11] Kolappa K., Henderson D.C., Kishore S.P. (2013). No physical health without mental health: Lessons unlearned?. World Health Organ..

[12] Ministry of Health, Labour and Welfare (2018). Annual Health, Labour and Welfare Report 2018.

[13] Ministry of Health, Labour and Welfare (2012). A Basic Direction for Comprehensive Implementation of National Health Promotion.

[14] Puschmann A.K., Drießlein D., Beck H., Arampatzis A., Catalá M.M., Schiltenwolf M., Mayer F., Wippert P.-M. (2020). Stress and Self-Efficacy as Long-Term Predictors for Chronic Low Back Pain: A Prospective Longitudinal Study. J. Pain Res..

[15] Theorell T. (2019). A long-term perspective on cardiovascular job stress research. J. Occup. Health.

[16] Dolcini-Catania L.G., Byrne M.L., Whittle S., Schwartz O., Simmons J.G., Allen N.B. (2020). Temperament and Symptom Pathways to the Development of Adolescent Depression. J. Abnorm. Child Psychol..

[17] Parwani R., Parwani S.R. (2014). Does stress predispose to periodontal disease?. Dent. Update.

[18] Ohrbach R., Michelotti A. (2018). The Role of Stress in the Etiology of Oral Parafunction and Myofascial Pain. Oral. Maxillofac. Surg. Clin. N. Am..

[19] World Health Organization (2018). ICD-11 for Mortality and Morbidity Statistics. Disorders Specifically Associated with Stress. http://id.who.int/icd/entity/991786158.

[20] Weathers F.W. (2017). Redefining posttraumatic stress disorder for DSM-5. Curr. Opin. Psychol..

[21] Abdallah C.G., Geha P. (2017). Chronic Pain and Chronic Stress: Two Slides of the Same Coin?. Chronic Stress.

[22] Iwasaki M., Sato M., Minagawa K., Manz M.C., Yoshihara A., Miyazaki H. (2015). Longitudinal relationship between metabolic syndrome and periodontal disease among Japanese adults aged ≥70 years: The Niigata Study. J. Periodontol..

[23] Kondo N., Kawachi I., Hirai H., Kondo K., Subramanian S.V., Hanibuchi T., Yamagata Z. (2009). Relative deprivation and incident function disability among older Japanese women and man: Prospective cohort study. J. Epidemiol. Community Health.

[24] Oates G.R., Juarez L.D., Hansen B., Kiefe C.I., Shikany J.M. (2020). Social Risk Factors for Medication Nonadherence: Findings from the CARDIA Study. Am. J. Health Behav..

[25] Klein E.M., Brähler E., Dreier M. (2016). The German version of the Perceived Stress Scale-psychometric characteristics in a representative German community sample. BMC Psychiatry.

[26] Kalmbach D.A., Abelson J.L., Arnedt J.T., Zhao Z., Schubert J.R., Sen S. (2019). Insomnia symptoms and short sleep predict anxiety and worry in response to stress exposure: A prospective cohort study of medical interns. Sleep Med..

[27] Zamkah A., Hui T., Andrews S., Dey N., Shi F., Sherratt R.S. (2020). Identification of Suitable Biomarkers for Stress and Emotion Detection for Future Personal Affective Wearable Sensors. Biosensors.

[28] Watanabe S., Li Y.S., Kawasaki Y., Kawai K. (2019). Workers’ Lifestyles and Urinary 8-hydroxydeoxyguanosine as an Oxidative Stress Marker. J. UOEH.

[29] Fueda Y., Matsuda F., Kataoka T. (2020). Assessment of noninvasive positive pressure ventilation in healthy young volunteers using salivary stress biomarkers. Future Sci. OA.

